# Using Object-Oriented Simulation to Assess the Impact of the Frequency and Accuracy of Mobility Scoring on the Estimation of Epidemiological Parameters for Lameness in Dairy Herds

**DOI:** 10.3390/ani14121760

**Published:** 2024-06-11

**Authors:** Rachel Clifton, Robert Hyde, Edna Can, Matthew Barden, Al Manning, Andrew Bradley, Martin Green, Luke O’Grady

**Affiliations:** 1 School of Veterinary Medicine and Science, University of Nottingham, Sutton Bonington Campus, Loughborough LE12 5RD, UK; robert.hyde3@nottingham.ac.uk (R.H.); edna.can2@nottingham.ac.uk (E.C.); matthew.barden@liverpool.ac.uk (M.B.); andrew.bradley@qmms.co.uk (A.B.); martin.green@nottingham.ac.uk (M.G.);; 2 Quality Milk Management Services Ltd., Cedar Barn, Wells BA5 1DU, UK; al.manning@qmms.co.uk

**Keywords:** lameness, dairy cattle, object-oriented simulation, prevalence, incidence, duration, mobility scoring, sensitivity, specificity, frequency

## Abstract

**Simple Summary:**

The standard method for monitoring lameness in U.K. dairy herds is mobility scoring. Data from mobility scoring can be used to estimate the proportion of cows in the herd that are lame (prevalence), the rate at which cows become lame (incidence), and how long cows remain lame (duration). It is unknown how the frequency and accuracy of mobility scoring impact the accuracy of measurement of these parameters. We developed a model to simulate lameness in a range of herd scenarios with different prevalences and durations of lameness. We used this model to understand how the frequency and accuracy of mobility scoring affected the accuracy of lameness parameters calculated from mobility scoring data. Our results showed that reduced accuracy of mobility scoring results in an over-estimation of lameness incidence and an under-estimation of lameness duration. This effect increased with more frequent scoring. Lameness prevalence and the average number of days to first lameness best identified lameness patterns when simulating monthly mobility scoring. We conclude that the frequency and accuracy of mobility scoring should be considered when using mobility scoring data to inform on lameness patterns on farms.

**Abstract:**

Mobility scoring data can be used to estimate the prevalence, incidence, and duration of lameness in dairy herds. Mobility scoring is often performed infrequently with variable sensitivity, but how this impacts the estimation of lameness parameters is largely unknown. We developed a simulation model to investigate the impact of the frequency and accuracy of mobility scoring on the estimation of lameness parameters for different herd scenarios. Herds with a varying prevalence (10, 30, or 50%) and duration (distributed around median days 18, 36, 54, 72, or 108) of lameness were simulated at daily time steps for five years. The lameness parameters investigated were prevalence, duration, new case rate, time to first lameness, and probability of remaining sound in the first year. True parameters were calculated from daily data and compared to those calculated when replicating different frequencies (weekly, two-weekly, monthly, quarterly), sensitivities (60–100%), and specificities (95–100%) of mobility scoring. Our results showed that over-estimation of incidence and under-estimation of duration can occur when the sensitivity and specificity of mobility scoring are <100%. This effect increases with more frequent scoring. Lameness prevalence was the only parameter that could be estimated with reasonable accuracy when simulating quarterly mobility scoring. These findings can help inform mobility scoring practices and the interpretation of mobility scoring data.

## 1. Introduction

Lameness represents a major health and welfare concern in dairy cows, with significant costs associated with treatment and reduced performance of affected animals [1]. Lameness is most frequently quantified in dairy herds using prevalence; prevalence estimates can be compared to target levels or national benchmarks and can be used to monitor lameness over time. However, the prevalence does not provide a sufficient description of lameness in dairy cows; the incidence and duration of lameness are also important to reveal the nature of lameness in a herd [2].

The calculation of the incidence and duration of lameness requires the collection of longitudinal data. In the U.K., mobility scoring using a four-point scale (0 = good mobility, 1 = imperfect mobility, 2 = impaired mobility, and 3 = severely impaired mobility) is the industry standard method for collecting lameness data on commercial farms [3], and this is commonly conducted monthly or quarterly. Eriksson, Daros [4] demonstrated that the frequency of scoring impacts the estimation of lameness incidence and recommended scoring every two weeks for longitudinal research studies. In a commercial setting, two-weekly scoring may be deemed unrealistic, and it is, therefore, essential to understand the impact of less frequent scoring on the accuracy of estimation of different lameness parameters.

A low intra- and interobserver agreement is frequently reported for mobility scoring reviewed by [5], and a low sensitivity for detecting mildly lame cows can result in the under-estimation of lameness prevalence [6]. How the accuracy of mobility scoring impacts on the estimation of the incidence and duration of lameness, and how this varies depending on the frequency of scoring, has also not been previously investigated.

The impact of the frequency and accuracy of mobility scoring on the estimation of lameness parameters may not be the same across all farms but may rather depend on the incidence, duration, and prevalence of lameness on each farm. To fully understand this, mobility scoring data from many farms with different levels of lameness would need to be obtained. Scoring would need to be conducted frequently (at least weekly) and over a relatively long time period. It is, therefore, unlikely that such data could be obtained. An alternative approach is to simulate data for a variety of herd scenarios. Simulation approaches have the advantage that they allow research questions to be answered without requiring large numbers of animals [7], and they provide a theoretical understanding of how different factors interact to influence an outcome of interest, e.g., [8,9]. In the context of livestock farming, they have most commonly been used to understand the economic consequences of different diseases or management interventions [10,11,12]. Simulation has not previously been used to investigate the estimation of lameness parameters from mobility scoring data.

The aim of our study was to develop and deploy a simulation model to investigate the impact of the frequency and accuracy of mobility scoring on the estimation of dairy herd lameness parameters including prevalence, incidence, and duration.

## 2. Materials and Methods

### 2.1. Object-Oriented Simulation Model Description

The object-oriented simulation model used in this study was developed as part of a wider project to create a real-time decision support system for dairy farms (REMEDY: REal tiME DairY; https://gtr.ukri.org/projects?ref=48717, accessed on 3 June 2024). One component of the REMEDY platform is a whole-farm model designed to allow accurate forecasting of key health, welfare, economic, and environmental outcomes and provide a framework for making informed farm management decisions. Below, we provide specific details relating to the simulation of lameness using the REMEDY simulation model; descriptions are provided with reference to the Overview, Design concepts, and Details (ODD) protocol [13]. The simulation model was coded in the Python programming language [14] utilising an object-oriented programming paradigm.

#### 2.1.1. Model Purpose

The proximate purpose of the lameness model was to simulate the dynamics of lameness in a dairy herd as a result of different incidences and durations of lameness cases. This allowed the simulation of a range of scenarios for which the availability of real-world data was limited. Our ultimate purpose was to explore how the accuracy of the estimation of herd-level lameness parameters varies with the frequency and accuracy of mobility scoring.

#### 2.1.2. Model Entities, Attributes, and Scales

**Figure 1:**
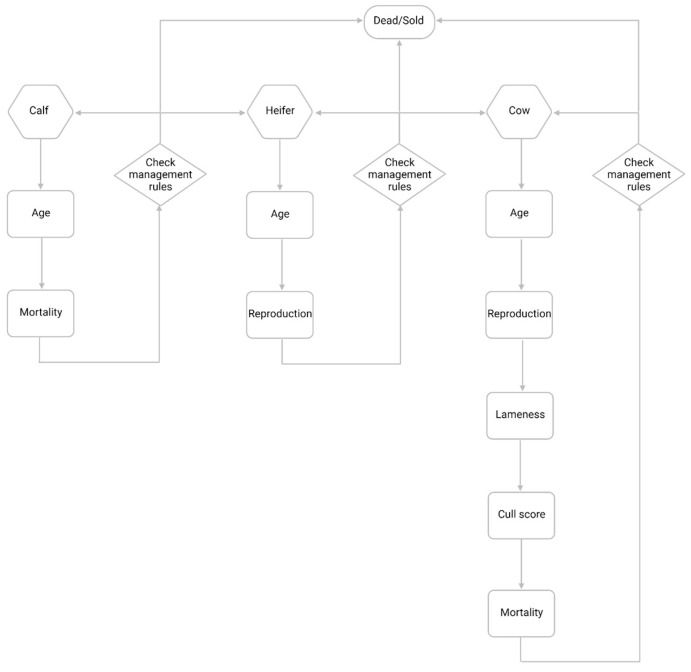
Process of simulating animals depending on management group at daily timesteps. Created with BioRender.com.

The model entities were the herd and cows within the herd. The simulation model was at cow level and was simulated in daily timesteps. The model did not represent the space or location of the animals. Real farm data were utilised for the instantiation of both herd and animal objects (see Section 2.1.3) including the following attributes: animal identifier, age, parity, lactational status, days in milk (DIM), reproductive status, lameness state, and current management group (full list in Table S1). The animals had a management group assigned, such as pre-weaned calf (age 0–41 days), weaned calf (age 42–364 days), breeding heifer (female, age ≥ 365 days, parity = 0), cow (female, age ≥ 365 days, parity ≥ 1), or bull (male, age ≥ 365 days), according to imported farm data (TotalVet, QMMS Ltd., Wells, UK). The functions being applied to each animal object were dependent upon the management group on a given day, and the animals could move through several management groups throughout their simulated lifetime (Figure 1).

#### 2.1.3. Process Overview and Scheduling

**Figure 2:**
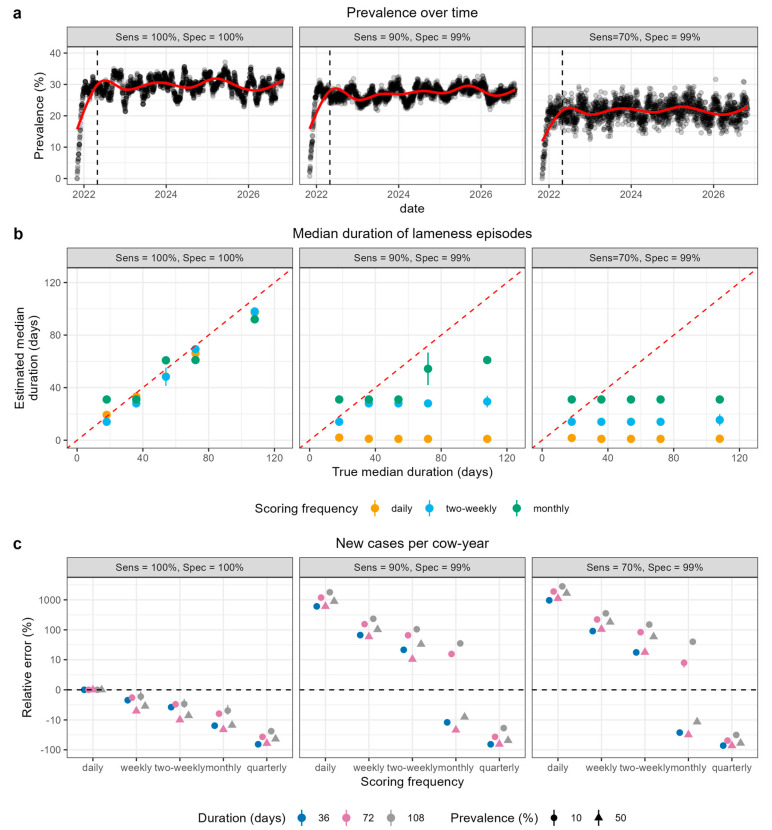
Impact of frequency and accuracy of mobility scoring on estimation of lameness parameters for simulated herd scenarios. (**a**) Daily prevalence estimates with varying accuracy of mobility scoring for one replicate simulation of a herd scenario with median prevalence = 30% and median duration = 72 days. Points show the estimated prevalence on each date, and the red line shows smoothed prevalence over time. The left panel shows “true” lameness, i.e., daily observations with 100% sensitivity and 100% specificity. The centre and right panels show estimated prevalence values based on mobility scoring with 99% specificity and 90% and 70% sensitivity, respectively. (**b**) Estimated median duration of lameness compared to “true” median duration. Herd scenarios with prevalence = 10% are shown as an example. Panels show different sensitivities and specificities of mobility scoring, and colours show different frequencies of mobility scoring. Points show the mean estimated duration across ten replicate simulations, and error bars show the standard deviation around this mean. Red dashed line shows x = y. (**c**) Variation in relative error in estimated new cases per cow-year with different frequencies and accuracies of mobility scoring for four example herd scenarios. The relative error was calculated as the difference between the estimated and true values divided by the true value and then converted to a percentage. A positive relative error indicates an over-estimation of the parameter, whereas a negative relative error indicates under-estimation. The true value was that calculated from the daily time-step data with 100% sensitivity and specificity. Colours show the true median duration, and shapes show the true median prevalence for each herd scenario. Panels show different sensitivities and specificities of mobility scoring. Points show the mean relative error across ten replicate simulations, and error bars show the standard deviation around this mean.

A “burn-in” period of 180 days was run to obtain a steady lameness prevalence (Figure 2a), and then, each simulation was run for a further 4.5 years (5 years total simulation time) to provide sufficient data for lameness parameter estimation. On the commencement of a simulation, data from a real, seasonal calving herd of 140 Holstein Friesian cows located in Southwest England was utilised to calculate key herd characteristics and populate the attributes of the animals currently on-farm. This herd was selected because the herd size is similar to the U.K. average [15], and high-quality data were available to calculate the herd characteristics and populate the animal attributes. Events were simulated for individual animals daily, dependent on the management group of each animal as shown in Figure 1. Reproduction and culling processes were defined to maintain the population dynamics and target herd size, as described below.

#### 2.1.4. Reproduction and Culling

The reproductive parameters including oestrus cycle length (mean 21 days, sd 2 days), gestation length (mean 282 days, sd 5 days), and heat detection and conception probabilities were simulated from the onset of puberty. Heat detection was assumed to have a sensitivity of 0.6 and a specificity of 0.999 [16]; the conception probability was 0.4 [17]; and the mean age of onset of puberty was 435 days based on imported farm data (see Supplementary Information for further details). The culling or sale of animals and mortality were simulated dependent on the management group. The culling of milking cows was based on animal age, pregnancy, and lameness status, i.e., older, non-pregnant cows with a history of lameness were more likely to be culled. The risk of mortality was based on mortality rates in the farm input data; for milking cows, the mortality varied by the stage of lactation (DIM < 50 days: 0.000613 day^−1^, 50–100 DIM: 0.000442 day^−1^). Further detail on the reproduction, culling/sale, and mortality sub-models is provided in the Supplementary Information. The lameness sub-model is described below.

#### 2.1.5. Lameness Model

Lameness was simulated for cows from their first calving and was simulated during both lactation and the dry period. The lameness attribute had two potential values, “sound” or “lame”. All cows were assigned as sound on initialisation of the simulation. Cows in the “sound” group became lame at a rate determined by the incidence parameter: each day, a random number in the interval (0, 1) was generated; if this was greater than the incidence parameter, the cows remained sound, otherwise their lameness state was updated to “lame”. Once a cow became lame, the duration of the lameness episode in days was set based on a gamma distribution with defined shape and scale parameters dependent on the duration input parameter (see Equations (2) and (3)). The end date for the lameness episode was then calculated as the current date plus the duration in days. For cows in the “lame” state, at each daily time step, the current date was compared to the end date for the lameness episode. Once the end date was reached, the lameness state was updated to “sound”.

The input parameters for the lameness model were the mean herd prevalence of lameness and the mean duration of lameness episodes. The daily incidence rate was then calculated as follows:

IR = (P/(1 − P))/D(1)

where IR was daily incidence rate, P was prevalence, and D was average duration in days.

The duration of lameness was modelled as a gamma distribution because the literature suggests that a small proportion of cows have much longer durations than the herd average [18,19]. The shape and scale parameters for the gamma distribution of the duration were calculated according to the following equations [20]:

Shape = D^2^/σ^2^ (2)

Scale = σ^2^/D (3)

where D was the average duration of lameness in days and σ was the standard deviation of the duration of lameness in days. The selection of input values for D and σ was as described below (Section 2.1.7). A minimum threshold was applied to prevent very short durations because we believed this would be unrealistic (Table 1).

#### 2.1.6. Stochasticity

The transitions from “sound” to “lame” were stochastic using pseudorandom number distributions to determine the outcome. The duration of the lameness episodes was also stochastic because it was drawn from a gamma distribution (Figure S1) for each lameness event. Stochasticity was used to enable the incidence and duration to vary around defined input parameters without needing to model the cause of the variability.

#### 2.1.7. Selection of Prevalence and Duration Input Parameters

The literature suggests that the mean within-herd prevalence of lameness in U.K. dairy herds is 30% [21,22]. We, therefore, selected three input prevalence values of 10%, 30%, and 50% to represent low-, average-, and high-prevalence herds (Table 1).

For the duration of lameness, the minimum values reported in the literature for U.K. herds are 14 days [19,23] whilst the median duration is suggested to be 4 weeks with an interquartile range of 2–7 weeks [24]. Lesion severity influences the recovery time, with severe lesions having longer recovery times [25]. We simulated lameness in our herd using different average duration values of 18, 36, 54, 72, and 108 days and with the minimum thresholds shown in Table 1. We included higher values than those reported in the literature, because without these, it was impossible to achieve the higher prevalence values reported [21,22] without extremely high incidence rates (Figure S2c).

There was little information from the literature to inform our choice of the standard deviation of duration (σ in Equations (2) and (3)). We, therefore, tested a variety of values for D/σ and found that a value of 2.5 produced distributions that best matched the authors’ clinical experience. The resulting gamma distributions for the duration are shown in Figure S1.

#### 2.1.8. Model Testing

The criteria for model usefulness were that the prevalence and duration of lameness calculated from the model outputs matched the values that were used as inputs.

### 2.2. Replicating Different Frequencies of Mobility Scoring

The model output was a .csv file containing the lameness state for every animal in the herd at every time step over ten repeat model runs. For each replicate model run, four additional datasets were created in R [26] by removing observations to mimic weekly, two-weekly, monthly, and quarterly mobility scoring. These additional intervals were selected because they are commonly used for mobility scoring in research studies or on commercial farms.

### 2.3. Varying the Sensitivity and Specificity of Mobility Scoring

To investigate the effect of varying the sensitivity and specificity of mobility scoring on the estimation of lameness parameters, we converted lame scores to sound scores with a probability equal to one minus the target sensitivity and converted sound scores to lame scores with a probability equal to one minus the target specificity. Therefore, to adjust for the sensitivity of mobility scoring, for each lame score, a random number was drawn from (0, 1), and if this number was greater than the specified sensitivity, the lame score was converted to a sound score, otherwise it remained a lame score. To adjust for specificity, the same process was applied, but sound scores were converted to lame scores. This process was carried out in R [26].

We used a range of values for the sensitivity and specificity of mobility scoring, so we could investigate relationships between sensitivity, specificity, frequency of mobility scoring, and herd lameness parameters. The sensitivity and specificity of mobility scoring for detecting lameness are not known and are likely to vary between observers and farms. Studies comparing mobility scores to the presence of lesions or claw pain report high specificity compared to sensitivity [27,28]. Extrapolating from this and the authors’ expertise, we defined specificity as either 95% or 99% whilst exploring sensitivity at values of 60, 70, 80, or 90%.

### 2.4. Calculating Lameness Parameters from Simulation Data

All calculations were performed in R [26]. The lameness parameters we investigated were prevalence and duration and three incidence-based parameters: new cases per cow-year, median days to first lameness event, and probability of remaining sound in the year after first calving. Lameness parameters were calculated for each dataset (daily, weekly, two-weekly, monthly, and quarterly scoring) as follows. The prevalence was observed to reach an equilibrium around 180 days into the simulation (Figure 2a); therefore, data from the first 180 days of each simulation were excluded as a burn-in period. The point prevalences and the incidence rate (new cases per cow-year) were calculated according to the equations below. The prevalence was then summarised as the median across all time points.

Point prevalence = Lame cows/Total cows × 100(4)

New cases per cow-year = Total new cases/At risk cow-years(5)

For the point prevalence, total cows refers to the number of cows in the “cow” management group (i.e., parity > 0) and, therefore, included in the lameness model on the date in question.

The duration of each lameness episode was calculated as the number of days between a cow first being scored lame and the first occasion that she was scored sound following the lameness episode. Left- and right-censored episodes (i.e., episodes that included the first or last time point) were excluded as the length of the episode could not be determined. The median duration was then calculated across all lameness episodes. The time from calving to the first lameness event analysis for first-parity animals was performed using the Kaplan–Meier method with the survival package [29] in R. Left-censored observations (i.e., individuals that were lame at the first time point) were excluded. The parameters reported were median days to first lameness event and one year probability of remaining sound.

### 2.5. Calculating Relative Error in Lameness Parameters

For each herd scenario, the true value of each lameness parameter was taken as the value calculated from the daily time-step data with 100% sensitivity and specificity. The “estimated” parameter values were those calculated when the frequency and/or accuracy of scoring was reduced. The relative error of the estimated parameters compared to the true value was calculated as the difference between the estimated and true values divided by the true value and then converted to a percentage. Finally, the mean and standard deviation of the relative error were calculated across the ten replicate simulations.

## 3. Results

We have provided example figures to illustrate the results we describe below. All the estimated lameness parameters and the relative error of these parameters compared to the true values are shown in Figures S4 and S5, respectively.

### 3.1. Model Testing

For each herd scenario, we compared model outputs from 4.5-year simulations to our input parameters. Our model accurately replicated a range of target prevalence and duration values (Example in Figure S2a,b). We confirmed that, after the initial burn-in period, the within-herd prevalence remained stable over the course of each simulation (e.g., Figure 2a).

Several of the scenarios resulted in incidence rates that we deemed to be unrealistic, particularly those with a high prevalence but low duration (Figure S2c). We decided to exclude scenarios that resulted in fewer than six new cases per cow-year from further analysis (n = 4 scenarios, Table 1).

### 3.2. Impact of Scoring Frequency on Estimation of Lameness Parameters

We initially investigated the impact of the scoring frequency on the estimation of lameness parameters without any adjustment for accuracy of scoring; therefore, we set the sensitivity and specificity of the mobility scoring to 100%. There was a clear effect of scoring frequency on accuracy of lameness parameter estimation under these conditions, and the nature of this relationship was different for different lameness parameters and different herd scenarios. The estimated median prevalence showed the least variation with scoring frequency, with no more than a 7% relative difference (i.e., the difference between the estimated and true value was 7% of the true value) between the estimated and true median values irrespective of the herd scenario. The estimated duration of lameness was the parameter most affected by the mobility scoring frequency because the estimated duration of the lameness episodes will always be a multiple of the length of the scoring interval. Therefore, the accuracy of the estimated duration increased with an increase in the frequency of scoring (the left panel of Figure 2b shows a comparison of daily, two-weekly, and monthly scoring).

In situations in which the lameness episodes were shorter than the interval between the scoring occasions, the incidence of lameness cases was under-estimated because cows could become lame and recover between mobility scoring occasions. This effect was evident in all three incidence parameters (new case rate, days to first lameness event, and probability of remaining sound in the first year); the left panel of Figure 2c shows an example for the new case rate. In this figure, a positive relative error indicates the over-estimation of a parameter, i.e., the estimated new case rate is greater than the true value.

### 3.3. Impact of Imperfect Mobility Scoring on Estimation of Lameness Parameters

In reality, mobility scoring is not 100% sensitive or specific. We, therefore, repeated our analysis using a range of sensitivity and specificity values for mobility scoring.

The relative error in the prevalence estimates across all simulated conditions ranged from <1% to 55%; i.e., the greatest difference between estimated and true prevalence was 0.55 times the size of the true value. The prevalence was increasingly under-estimated with decreasing sensitivity and increasingly over-estimated with decreasing specificity (Figure S5) as would be expected [30]. Figure 2a shows an example of this for herd scenario p30_d72: in the right panel (sensitivity = 70% and specificity = 99%), the smoothed prevalence over time is stable at about 22% rather than at the true prevalence of 30%.

The relative error in the estimates of incidence parameters across all simulated conditions ranged from <1% to 7479%, 783%, and 4300% for new case rate, median days to first lameness, and probability of remaining sound in the year after first calving, respectively (Figure S5). The impact of the sensitivity and specificity on the estimation of incidence parameters varied with the scoring frequency and herd scenario (an example of this is shown for median time to first lameness in Figure 2c). The most notable effect was the over-estimation of incidence with daily, weekly, and two-weekly scoring; the relative error was the highest for the new case rate estimated from daily scoring. With monthly scoring, the effect of reducing the sensitivity and specificity was dependent on the herd scenario, and both under- and over-estimation of incidence occurred (shown by positive and negative relative errors in the new case rate for monthly scoring in the middle and right panel of Figure 2c).

The relative error in the estimates of median duration across all simulated conditions ranged from <1% to 384% and varied with the scoring frequency and herd scenario (Figure S5). The reduced sensitivity and/or specificity of scoring caused the estimated duration of lameness to tend towards the length of scoring interval (Figure 2b and Figure S3). Therefore, the most marked under-estimation of duration occurred for scenarios with long durations and daily scoring. In contrast, there was minimal impact of reducing the sensitivity and specificity on the estimates of duration from quarterly scoring because these were already equal to the length of the scoring interval (Figure S5).

### 3.4. Differentiation between Scenarios with the Same Prevalence

**Figure 3:**
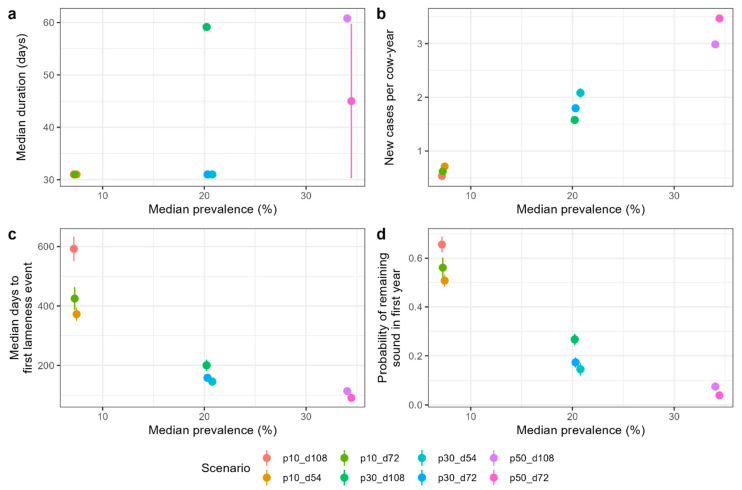
Differentiation between herd scenarios using lameness parameters calculated from imperfect monthly mobility scoring. The lameness parameters, (**a**) estimated median duration, (**b**) estimated new cases per cow-year, (**c**) estimated median days to first lameness event in heifers, and (**d**) estimated probability of remaining sound in the year after first calving, are shown dependent on the estimated median prevalence of lameness (x axis) and herd scenario (point colour). Points show the mean estimated parameter across ten replicate simulations and error bars show the standard deviation in the y-axis parameter around this mean. Specificity = 99%, sensitivity = 70%, and scoring interval = monthly. For scenarios shown in the legend, p indicates prevalence (%) and d indicates duration (days).

Whilst the prevalence of lameness was the parameter least affected by the frequency or accuracy of mobility scoring, the prevalence is less useful to inform on the patterns of lameness on a farm than the other parameters investigated. We, therefore, examined which parameters facilitated the differentiation of herd scenarios with the same prevalence but different incidences and durations. To achieve this, we replicated monthly scoring with a sensitivity of 70% and a specificity of 99% as a reasonable representation of mobility scoring on commercial farms. Figure 3 shows the estimated incidence and duration parameters for the different herd scenarios dependent on their estimated prevalence to visualise whether scenarios that appear similar in terms of estimated prevalence (x axis) could be separated in terms of the y-axis parameter under these conditions. It is clear from Figure 3a that, in this situation, the estimated median duration of lameness did not differentiate between herd scenarios, with most estimated median duration values equal to the scoring interval (~30 days). The three incidence parameters provided similar differentiation between scenarios with the same prevalence, although the magnitude of the difference between scenarios was relatively small (shown by the separation of points with respect to the y-axis in Figure 3b–d).

## 4. Discussion

In this study, we developed an object-oriented simulation model of lameness in dairy herds to understand how the frequency and accuracy of mobility scoring impact on the estimation of a variety of lameness parameters. Eriksson, Daros [4] previously provided evidence that the frequency of mobility scoring affected the estimation of lameness incidence; however, our simulation-based approach allowed us to perform more detailed investigations over a broad range of scenarios. This allowed us to establish the relationships between the prevalence and duration of lameness in a herd and the frequency and accuracy of mobility scoring. Below, we discuss the practical implications of our findings and the choices that were made during model development.

### 4.1. Practical Implications of Results

The relationship between the frequency of mobility scoring and the true duration of lameness episodes was a key determinant of the accuracy of the evaluation of lameness parameters. Substantial inaccuracies occurred in the estimates of all lameness parameters except the prevalence when the true duration of lameness was shorter than the interval between mobility scores. The median duration of lameness is reported as 4 weeks [24], and our data highlight that, on farms with an average lameness prevalence (30%), median a duration <30 days cannot occur without very high incidence rates (Table 1 and Figure S2). We would, therefore, suggest that a minimum of monthly scoring is required for the meaningful interpretation of lameness data in a commercial setting. From our data, two-weekly or monthly scoring appears optimum for assessing the key metrics. Quarterly scoring is likely to under-estimate the incidence, time to first lameness, and the proportion remaining sound in lactation one and over-estimate the lameness duration.

Accounting for uncertainty in the mobility scoring data by changing the sensitivity and specificity greatly impacted on the accuracy of all the estimated lameness parameters with daily, weekly, and two-weekly scoring. Even optimistic estimates of sensitivity resulted in estimated duration values equal to the length of the scoring interval, which were, therefore, meaningless. Caution should be exercised in interpreting duration values calculated from mobility scoring data, particularly if the estimated median duration of lameness is observed to equal to the length of the scoring interval.

Whilst, in this study, the daily data were largely included as a baseline comparison for the other measures, the findings relating to sensitivity and specificity are relevant considering the increasing use of sensor-based technologies that report in real time [31]. The existing research has focused on the ability of the data from sensors to predict mobility score, e.g., [32,33] rather than on the impact of the frequency at which data are collected. Our results showed that, with daily data, even small decreases in the sensitivity and specificity resulted in a large over-estimation of incidence and an under-estimation of duration. With this type of data, it will, therefore, be necessary to implement some form of correction for this effect, and the approach we have developed will be valuable to investigate this further.

In summary, none of the parameters we studied were completely robust to the effects of the frequency and accuracy of mobility scoring. It is unlikely that mobility scoring will be carried out more frequently than monthly for most U.K. herds, and assuming an imperfect sensitivity and specificity of mobility scoring, our results indicate that the assessment of prevalence alongside a measure of incidence such as time to first lameness event may be the best way to benchmark and monitor lameness.

### 4.2. Model Development and Input Choices

In this study, we took a theoretical approach, whereby we simulated “true” lameness data, and various choices were made in building our lameness model. There was limited information available in the literature to inform the parameterisation of the model, and therefore, we based our input values for the duration and prevalence on a combination of the available literature and our expertise. Our objective was to explore the interaction between our input parameters and the frequency of mobility scoring rather than to recreate specific scenarios, and therefore, we explored a range of values for each input. We also explored a range of possible values of sensitivity and specificity to provide a better understanding of the relationship between the estimated lameness parameters and the accuracy of mobility scoring in different scenarios.

We decided to model all lameness as being of a similar type with the same epidemiological parameters; therefore, we did not attempt to recreate scenarios in which multiple different aetiologies (e.g., different foot lesion types) occur together within a herd. This was to ensure a clear demonstration of the relationships between our input parameters and the frequency of mobility scoring. It has previously been suggested that the presence of both infectious and claw horn lesions within a herd would likely create a bimodal distribution for duration [4]; therefore, the results from the current study could be interpreted with respect to each of these distributions separately. Our model does have the capability to include multiple prevalence and duration inputs; therefore, this could be investigated further in future studies.

The limitations of this study include that we did not model associations between the incidence of lameness and either parity or days in milk although these have been reported [34,35,36]. We did not model lameness in the dry period differently to that occurring during lactation, and we did not include different parameters for first and recurrent cases. We also did not include changes in incidence and prevalence over time, such as seasonal changes. Our model could be expanded to include these features in future work.

Finally, we replicated the sensitivity and specificity randomly rather than allowing an increased probability of false positives or negatives for some observations compared to others. In reality, the sensitivity and specificity are likely to be better for MS3 (severely impaired mobility) than for MS2 (impaired mobility). Previous evidence shows that poor agreement in mobility scoring occurs between cows with imperfect and impaired mobility, with cows that are severely lame being unlikely to be classified as sound [37]. In most U.K. herds, there are only a small proportion of cows that are severely lame at any one time point with most cows being classified as either MS1 (imperfect mobility) or MS2 (impaired mobility) [23,24,38]. We, therefore, believe that the model we created was a reasonable representation for our purpose. It would be possible to expand the model to include different severities of lameness; however, an increase in complexity would be required to model the transitions between multiple different states, and there are little data available to inform the parametrisation of such a model.

## 5. Conclusions

In this study, we developed a novel approach for the simulation of lameness within a dairy herd and demonstrated the value of this approach for understanding how the measurement of lameness impacts on the accuracy of the estimated epidemiological parameters. Our findings highlight that accurate estimation of the lameness parameters other than the prevalence cannot be achieved with quarterly mobility scoring. In addition, an over-estimation of lameness incidence and an under-estimation of duration occur with imperfect mobility scoring, and this effect increases with more frequent scoring. We propose that, on commercial farms using monthly mobility scoring, prevalence together with a measure of incidence may provide the best way to understand lameness patterns.

## Figures and Tables

**Table 1:** Simulated scenarios by input prevalence and duration parameters used in the simulation.

Input Duration (Minimum Threshold)	Input Prevalence		
	10%	30% ^1^	50% ^1^
18 (14) days	p10_d18	Excl	Excl
36 (30) days	p10_d36	p30_d36	Excl
54 (30) days	p10_d54	p30_d54	Excl
72 (30) days	p10_d72	p30_d72	p50_d72
108 (60) days	p10_d108	p30_d108	p50_d108

^1^ Excl = combinations of prevalence and duration that were excluded from analysis because they resulted in >6 new cases per cow-year.

## Data Availability

The simulated data supporting the conclusions of this article will be made available by the authors on request. The real farm data used to populate the simulation model are not readily available due to privacy reasons.

## Supplementary Materials

The following supporting information can be downloaded at https://www.mdpi.com/article/10.3390/ani14121760/s1: Supplementary methods: Description of object-oriented simulation sub-models for reproduction, culling/sale of animals from the herd, and mortality; Table S1: Summary of attributes for animal objects in the REMEDY object-oriented simulation model; Figure S1: Distributions for duration of lameness episodes used in simulation models; Figure S2: Results of model testing for an object-oriented simulation model of lameness in a dairy cow herd; Figure S3: Distribution of duration of simulated lameness episodes with different frequency and accuracy of mobility scoring for herd scenario with median prevalence of lameness of 30% and median duration of lameness of 72 days; Figure S4: Variation in estimated lameness parameters with different frequencies and accuracies of mobility scoring for all herd scenarios; Figure S5: Variation in relative error in estimated lameness parameters with different frequencies and accuracies of mobility scoring for all herd scenarios.
