# Supplementary material for: Using Object-Oriented Simulation to Assess the Impact of the Frequency and Accuracy of Mobility Scoring on the Estimation of Epidemiological Parameters for Lameness in Dairy Herds
Source: Animals (Basel). 2024 Jun 11;14(12):1760. doi: 10.3390/ani14121760 (PMC11200474; doi:10.3390/ani14121760)
Supplement: Supplementary file 1 [file animals-14-01760-s001.zip › Supplementary figures.pdf]

Supplementary figures

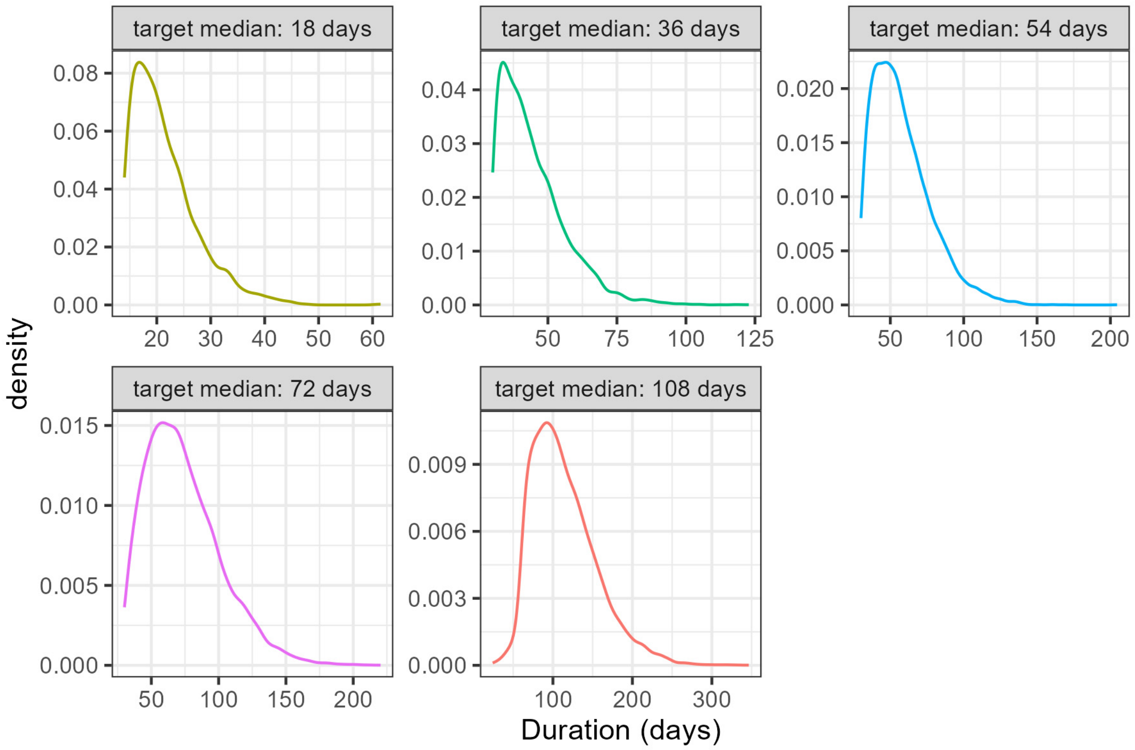

Figure S1 Distributions for duration of lameness episodes used in simulation models. Duration of each individual lameness episode within a simulation was drawn from the distribution shown for the target median duration used in the simulation.

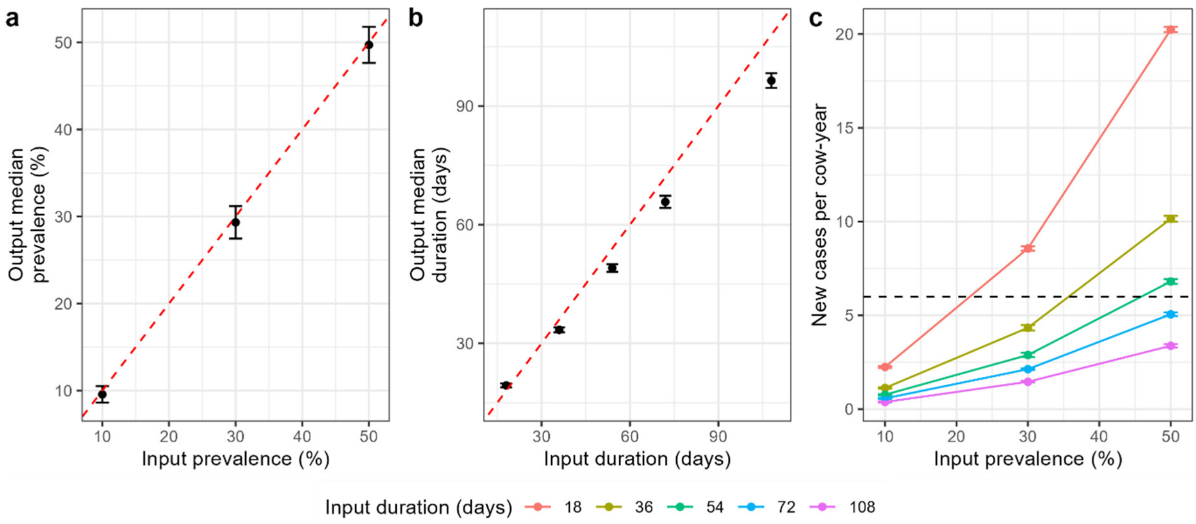

Figure S2 Results of model testing for an object-oriented simulation model of lameness in a dairy cow herd. Figure shows (a) median lameness prevalence estimated from simulation output compared to the input prevalence parameter used in the simulation, (b) median duration of lameness episodes estimated from simulation output compared to the input duration parameter used in the simulation, (c) new cases per cow-year estimated from simulation output by input prevalence and input duration parameters used in the simulation. All parameters were calculated from daily lameness states outputted by the simulation with 100% sensitivity and specificity. Points show mean across ten replicate simulations and error bars show standard deviation around this mean.

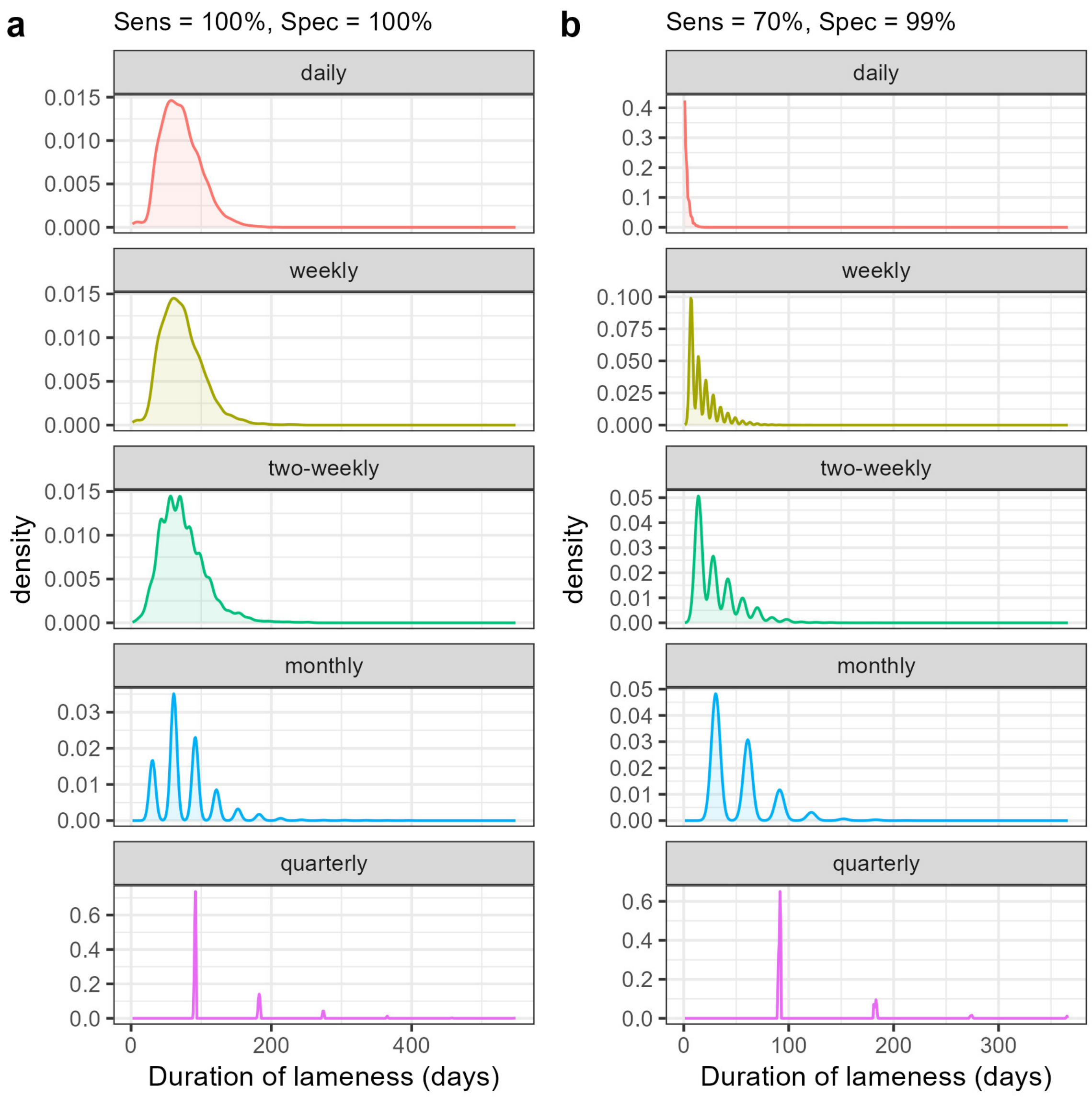

Figure S3 Distribution of duration of simulated lameness episodes with different frequency and accuracy of mobility scoring for herd scenario with median prevalence of lameness of 30% and median duration of lameness of 72 days. a Durations estimated replicating data from mobility scoring with 100% sensitivity and specificity, **b** Durations estimated replicating data from mobility scoring with 70% sensitivity and 99% specificity. The tendency for estimated duration of lameness episodes to tend towards the length of scoring interval when accuracy of mobility scoring is reduced is evident in panel b.

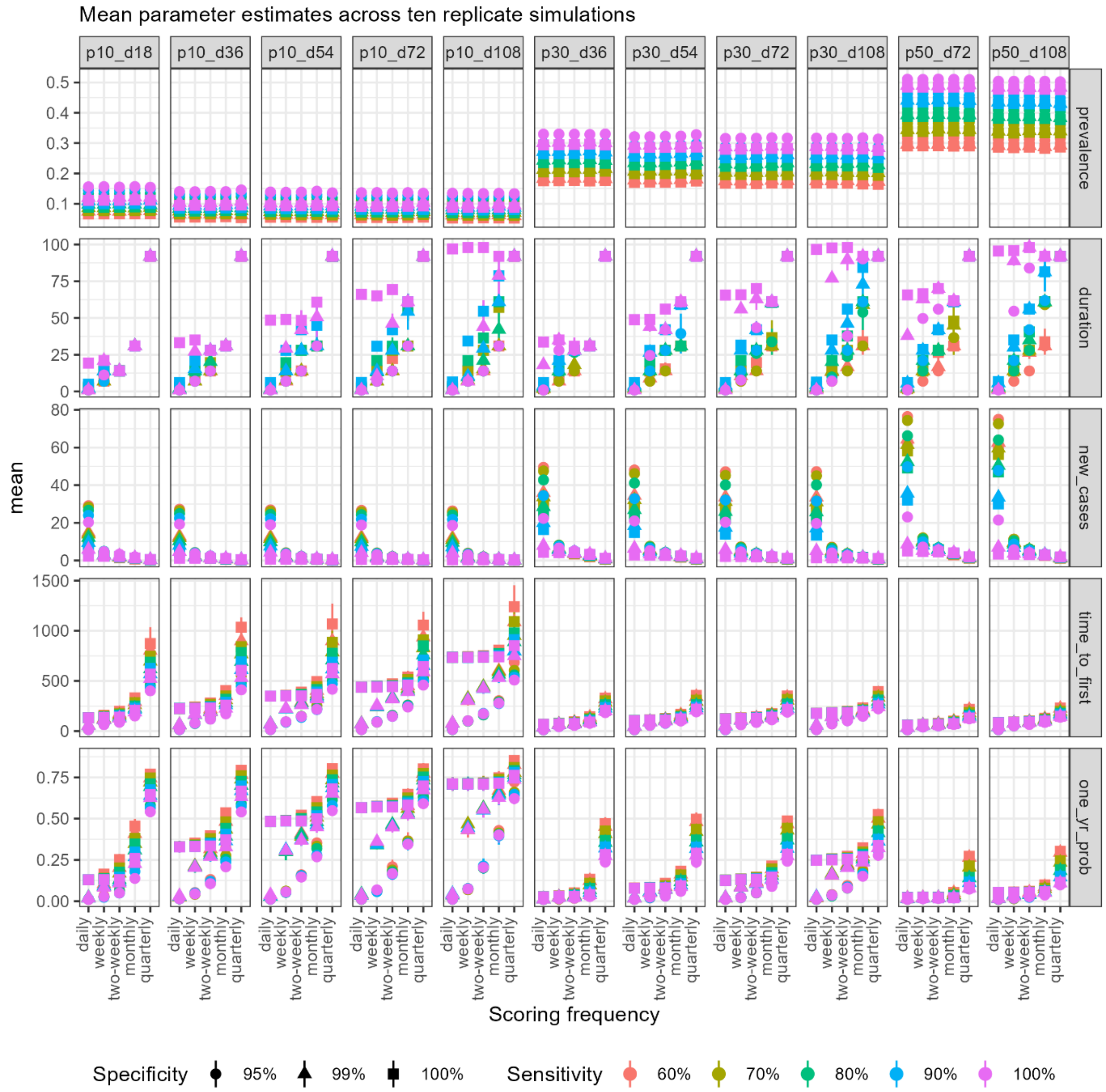

Figure S4 Variation in estimated lameness parameters with different frequency and accuracy of mobility scoring for all herd scenarios. Colours show sensitivity and shapes show specificity. Columns show different herd scenarios and rows show different lameness parameters. Points show the mean parameter value across ten replicate simulations and error bars show the standard deviation around this mean.

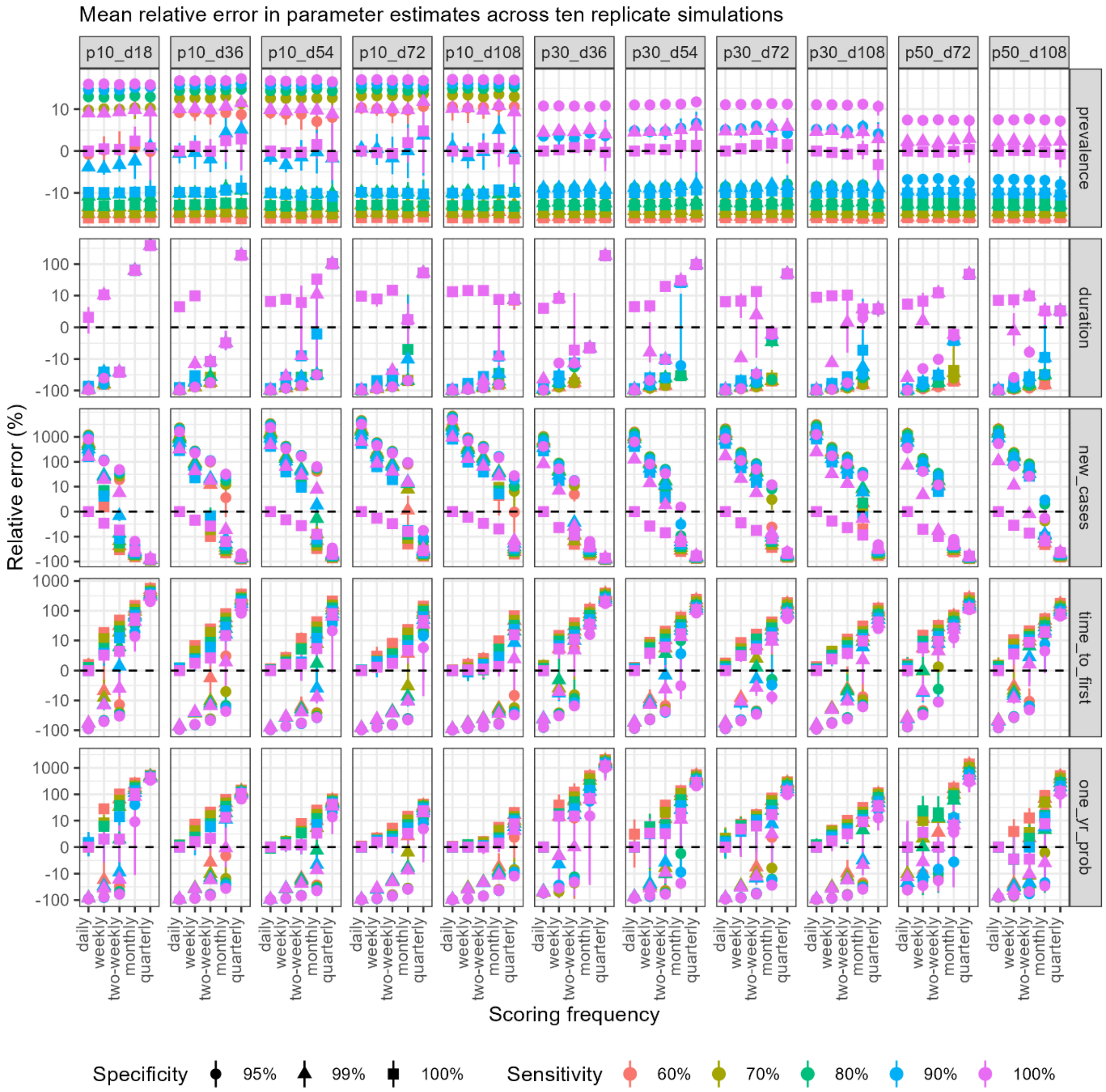

Figure S5 Variation in relative error in estimated lameness parameters with different frequency and accuracy of mobility scoring for all herd scenarios. Relative error was calculated as the difference between the estimated and true values divided by the true value and then converted to a percentage. True value was that calculated from daily time step data with 100% sensitivity and specificity. Colours show sensitivity and shapes show specificity. Columns show different herd scenarios and rows show different lameness parameters. Points show the mean relative error across ten replicate simulations and error bars show the standard deviation around this mean.
