# Supplementary material for: Using Object-Oriented Simulation to Assess the Impact of the Frequency and Accuracy of Mobility Scoring on the Estimation of Epidemiological Parameters for Lameness in Dairy Herds
Source: Animals (Basel). 2024 Jun 11;14(12):1760. doi: 10.3390/ani14121760 (PMC11200474; doi:10.3390/ani14121760)
Supplement: Supplementary file 1 [file animals-14-01760-s001.zip › Supplementary methods.pdf]

Description of object-oriented simulation sub-models for reproduction, culling/sale of animals from the herd and mortality

Reproduction

Reproductive parameters were based on published research and followed a mechanistic model of production (Ruelle *et al.*, 2015) where puberty, oestrus, insemination, conception, gestation, calving are all modelled in a cyclical process.

Reproductive parameters were simulated from onset of puberty, defined for each animal by sampling from a normal distribution around the median age at first service of heifers in the input farm data ($\bar{X}$ = 435, σ = 435 * 0.15). Oestrus cycles were simulated with durations sampled from a normal distribution ($\bar{X}$ = 21, σ = 2), and oestrus detection was dependent on sensitivity and specificity of heat detection of 0.6 and 0.999 respectively. Conception was stochastic with animals having probability of 0.4 of conceiving to each service. If conception occurred, calf sex was drawn from a binomial distribution with probability = 0.5 of being female, and calf breed type (dairy or beef) was drawn from a binomial distribution with probability of dairy equal to twice the herd replacement rate. Pregnant animals remained pregnant for a defined gestation length ($\bar{X}$ = 282, σ = 5) and pregnancy loss was tested daily and occurred stochastically with a probability of 0.03/282. Cows regained cyclicity a given number of days after pregnancy loss ($\bar{X}$ = 21, σ = 2). If pregnancy loss did not occur, animals ultimately calved, resulting in new animal object instances being instantiated. Cows regained cyclicity a given number of days after calving ($\bar{X}$ = 21, σ = 2) and were eligible to be served based on a voluntary waiting period of 42 days.

Culling/sale of animals from the herd

Culling or sale of milking cows, heifers and calves occurred monthly within the simulation.

Milking cows

Milking cows were selected for culling based on a cull score attribute. Culling points were allocated based on parity, fertility and lameness as shown in Table S2. Cull scores for fertility and lameness were arbitrary whereas those for parity were determined based on survival analysis of input farm data. Cumulative probability of exit by parity was determined and scaled to a maximum value of 100 to give cull points. Cull scores were calculated daily for each individual as the sum of all culling points.

Once monthly, the number of milking cows was compared to a target maximum herd size defined by the maximum number of milking cows during the last year for the input herd (133 cows). If the simulated herd size exceeded the target herd size, cows were grouped by cull score and then groups culled in order of descending cull score until the target herd size was reached. If there were more cows in a cull score group than required to achieve target herd size, the required number of cows were randomly selected from the group.

Table S2 Culling points attributed to milking cows based on parity, fertility and lameness in the REMEDY object-oriented simulation model

Criteria	Number of cull points
Parity 2-3	26
Parity 4-5	58
Parity ≥ 6	100
Not pregnant and > 100 DIM	25
Not pregnant and > 200 DIM	50
Not pregnant and > 300 DIM	100
Lame	40, reduced to 20 on recovery

Heifers

Removal of heifers occurred monthly. Heifers that had not become pregnant after 180 days of being eligible for breeding were randomly selected for removal to maintain a target number of replacements of 30. This was determined based on the median number of first parity animals over the previous year of imported farm data.

Calves

Male and beef calves older than 492 days were removed. This threshold was calculated as the median exit age for male animals in the imported farm data.

Mortality

Mortality occurred stochastically based on rates calculated from farm input data for the following groups: perinatal calves (age <= 2days; 0.074409/day), pre-weaned calves (age 3-41 days; 0.001358/day), weaned calves (age 42-364 days; 0.000203/day), early post calving cows (DIM < 50days; 0.000613/day) and later post calving cows (50-100 DIM; 0.000442/day).

To calculate mortality rates from farm input data, mortality rate for each age group for each year of entry to the herd was calculated as:

Mortality rate = (Total exits/Total animals)/number of days in age range

Where:

Total exits = number of animals that died or were sold whilst in the age range of interest (animals sold were included because our main purpose was to determine the rate of exit from the farm and specific reasons for sale could not be identified from farm data).

Total animals = number of animals born during that year

The median mortality rate across all years of entry was then used as the overall mortality rate for that age group.
