# Supplementary material for: Using Object-Oriented Simulation to Assess the Impact of the Frequency and Accuracy of Mobility Scoring on the Estimation of Epidemiological Parameters for Lameness in Dairy Herds
Source: Animals (Basel). 2024 Jun 11;14(12):1760. doi: 10.3390/ani14121760 (PMC11200474; doi:10.3390/ani14121760)
Supplement: Supplementary file 1 [file animals-14-01760-s001.zip › Table S1.pdf]

Table S1. Summary of attributes for animal objects in the REMEDY object-oriented simulation model

Variable	Entity	Possible values
Animal ID	All	Character string
Age (days)	All	Numeric
Date of birth	All	Date
Sex	All	Female<br>Male
Management group	All	Pre-weaned calf<br>Weaned calf<br>Heifer<br>Cow<br>Bull
Breed type	All	Dairy<br>Beef
Calf sex	Cows and heifers	Female<br>Male
Calf breed type	Cows and heifers	Dairy<br>Beef
Pregnancy status	Cows and heifers	Pregnant<br>Non-pregnant
Last service date	Cows and heifers	Date
Last calving date	Cows	Date
Next calving date	Cows and heifers	Date
Next oestrus date	Cows and heifers	Date
Parity	Cows and heifers	Integer
Eligible for breeding	Cows and heifers	True<br>False
Puberty date	Heifers	Date
Last conception date	Cows and heifers	Date
Days in calf	Cows and heifers	Numeric
Days in milk	Cows	Numeric
Dry	Cows	True<br>False
Lame state	Cows	Sound<br>Lame
Lame duration (days)	Cows	Numeric
Lame end date	Cows	Date
Lame cull score	Cows	Numeric
Cull score	Cows	Numeric
